# Supplementary material for: Isolation, Molecular Identification and Mycotoxin Profile of Fusarium Species Isolated from Maize Kernels in Iran
Source: Toxins (Basel). 2019 May 24;11(5):297. doi: 10.3390/toxins11050297 (PMC6563283; doi:10.3390/toxins11050297)
Supplement: Supplementary file 1 [file toxins-11-00297-s001.pdf]

Supplementary Materials: Isolation, Molecular Identification and Mycotoxin Profile of Fusarium Species Isolated from Maize Kernels in Iran

Maryam Fallahi, Hossein Saremi, Mohammad Javan-Nikkhah, Stefania Somma, Miriam Haidukowski, Antonio Francesco Logrieco and Antonio Moretti

Table S1. Fumonisin B_1 (FB_1), B_2 (FB_2) and B_3 (FB_3) production by *F. verticillioides* and *F. proliferatum* strains from Iranian maize kernels.

Species	Iranian Province	Strain ITEM code	FB_1 (μg/g)	FB_2 (μg/g)	FB_3 (μg/g)	Total FBs (μg/g)
F. verticillioides	Qazvin	18034	461	n.d.	n.d.	461
	Qazvin	18037	783	n.d.	n.d.	783
	Qazvin	18039	509	n.d.	n.d.	509
	Qazvin	18041	544	n.d.	n.d.	544
	Qazvin	18043	819	n.d.	n.d.	819
	Qazvin	18045	1103	n.d.	n.d.	1103
	Qazvin	18049	526	n.d.	n.d.	526
	Qazvin	18052	164	n.d.	n.d.	164
	Qazvin	18054	568	n.d.	n.d.	568
	Qazvin	18056	415	n.d.	n.d.	415
	Qazvin	18057	611	n.d.	n.d.	611
	Qazvin	18059	526	n.d.	n.d.	526
	Qazvin	18061	83	n.d.	n.d.	83
	Qazvin	18063	526	n.d.	n.d.	526
	Qazvin	18065	221	n.d.	n.d.	221
	Khuzestan	18066	307	n.d.	n.d.	307
	Khuzestan	18069	291	n.d.	n.d.	291
	Khuzestan	18072	507	n.d.	n.d.	507
	Khuzestan	18074	236	n.d.	n.d.	236
	Khuzestan	18076	568	n.d.	n.d.	568
	Khuzestan	18078	444	n.d.	n.d.	444
	Khuzestan	18081	595	n.d.	n.d.	595
	Khuzestan	18083	553	n.d.	n.d.	553
	Khuzestan	18087	2232	n.d.	n.d.	2232
	Khuzestan	18089	580	n.d.	n.d.	580
	Khuzestan	18092	280	n.d.	n.d.	280
	Khuzestan	18094	596	n.d.	n.d.	596
	Khuzestan	18096	273	n.d.	n.d.	273
	Fars	18099	617	n.d.	n.d.	617
	Fars	18101	572	n.d.	n.d.	572
	Fars	18104	216	n.d.	n.d.	216
	Fars	18108	704	n.d.	n.d.	704
	Fars	18110	1446	n.d.	n.d.	1446
	Fars	18112	411	n.d.	n.d.	411
	Fars	18114	444	n.d.	n.d.	444

	Fars	18116	423	n.d.	n.d.	423
	Fars	18118	472	n.d.	n.d.	472
	Fars	18120	786	n.d.	n.d.	786
	Fars	18122	1027	n.d.	n.d.	1027
	Fars	18124	694	n.d.	n.d.	694
	Fars	18127	399	n.d.	n.d.	399
	Fars	18129	449	n.d.	n.d.	449
	Fars	18131	505	n.d.	n.d.	505
	Ardabil	18133	386	n.d.	n.d.	386
	Ardabil	18135	255	n.d.	n.d.	255
	Ardabil	18137	374	n.d.	n.d.	374
	Ardabil	18139	430	n.d.	n.d.	430
	Ardabil	18141	147	n.d.	n.d.	147
	Ardabil	18143	488	n.d.	n.d.	488
	Ardabil	18145	514	n.d.	n.d.	514
	Ardabil	18147	442	n.d.	n.d.	442
	Ardabil	18149	453	n.d.	n.d.	453
	Ardabil	18151	415	n.d.	n.d.	415
	Ardabil	18153	475	n.d.	n.d.	475
	Ardabil	18155	359	n.d.	n.d.	359
	Ardabil	18157	552	n.d.	n.d.	552
	Ardabil	18159	428	n.d.	n.d.	428
	Ardabil	18161	479	n.d.	n.d.	479
	Khuzestan	18172	379	n.d.	n.d.	379
	Khuzestan	18174	387	n.d.	n.d.	387
	Golestan	18233	537	n.d.	n.d.	537
	Golestan	18235	304	n.d.	n.d.	304
	Golestan	18238	246	n.d.	n.d.	246
	Golestan	18240	451	n.d.	n.d.	451
	Golestan	18241	329	n.d.	n.d.	329
	Golestan	18242	79	n.d.	n.d.	79
	Golestan	18243	416	n.d.	n.d.	416
F. proliferatum	Qazvin	18036	78	5.0	6.6	90
	Qazvin	18040	15	n.d.	n.d.	15
	Qazvin	18051	187	0.4	0.5	187
	Qazvin	18163	45	3.4	1.2	49
	Qazvin	18177	1860	465.7	9.7	2335
	Qazvin	18182	110	1.7	n.d.	112
	Qazvin	18187	131	1.7	n.d.	133
	Qazvin	18189	1	n.d.	n.d.	1
	Fars	18097	201	n.d.	n.d.	201
	Fars	18098	273	0.5	0.4	274
	Fars	18214	86	n.d.	n.d.	86
	Ardabil	18222	169	2.9	1.1	173
	Golestan	18225	351	n.d.	n.d.	351
	Golestan	18226	274	n.d.	n.d.	274
	Golestan	18227	218	24.8	10.6	253
	Golestan	18228	97	0.6	n.d.	98
	Golestan	18231	187	1.3	0.5	188

Golestan	18234	54	0.3	n.d.	55
Alborz	18244	195	3.0	2.3	201
Alborz	18245	97	1.3	0.7	99
Alborz	18247	5	1.4	1.4	8
Alborz	18248	142	n.d.	n.d.	142
Alborz	18255	92	n.d.	n.d.	92
Alborz	18259	486	n.d.	n.d.	486
Alborz	18260	205	2.6	n.d.	208
Esfahan	18267	66	0.3	n.d.	67

n.d.: not detected.
